# Implementation of an Experiential Service-Learning Course in Biomedical Engineering Design for Undergraduate Students

**DOI:** 10.1007/s43683-022-00103-1

**Published:** 2023-01-23

**Authors:** Justyn Jaworski, Michael Cho

**Affiliations:** Department of Bioengineering, University of Texas at Arlington, Arlington, TX, USA

**Keywords:** Service learning, Community engagement, Bioengineering, Design course, Undergraduate, Team-based

## Abstract

The unique characteristics of the training needed for today’s biomedical engineers can represent a challenge in curriculum design. Practical experiential learning for biomedical engineering undergraduates is important to prevent under-developed professional skills. In this teaching tips article, we provide an example of how to incorporate experiential learning into the biomedical engineering curriculum to address the need for undergraduates to gain the desired skillsets to serve as the next generation of leaders in engineering, medicine, and business all through the lens of civic engagement. Here we outline our implementation of a recently developed service-learning course for our sophomore students that allows introduction of biomedical engineering discipline-specific design process early on in their undergraduate studies. Student teams work to design, build, and test novel devices to solve the unmet need of community partners, and in doing so, the course prepares students in developing technologies that not only address public health needs but that are also embraced by the community. This course in team-based design can help train students in analyzing real world problems for needs-based biomedical engineering through projects identified by interaction with community partners. Providing specifics of how this course was implemented as well as our reflection on student learning, we offer an analysis of the areas of success, a discussion of how interactions with community partners benefits the student professional skills development, and considerations regarding implementation. Here we highlight the ability of this course to exercise students’ social awareness in the design of technologies to improve society by addressing the genuine needs of community partners.

## CHALLENGE STATEMENT

In addition to technical know-how and analytical skills, it is imperative to the success of new biomedical engineering graduates entering the workforce to have refined professional skills (shifting from the prior terminology often used of ‘soft-skills’^3^) such as teamwork, critical thinking, communication, social responsibility, and organizational skills like prioritization/time management. Industry perspectives have highlighted that such professional skills are not being sufficiently nurtured among the various skill-sets provided to engineering graduates, which results in low retention rates of new hires.^8,11,12,14,15,17^ Problem-based group learning has been reported to significantly improve certain professional skills including the ability to work effectively on a team and overcome communication conflicts.^5^ Traditional lecture-based engineering courses can provide surrogate experiences to help develop professional skills to some extent through student-directed presentations, writing assignments, or short projects embedded as a component of the course; however, team-based design courses are naturally poised to provide students with continuous engagement in several areas for building professional skills. Authentic engineering learning environments utilizing real-world client-based problems provide the greatest source for professional skills development.^18^ An ongoing challenge is sourcing a fresh set of open-ended, clinically relevant problems each semester for which student teams can design, build, and test engineering solutions. In this teaching tip, we introduce an approach that addresses the challenge of professional skills development and sourcing of real-world design projects through the use of experiential service learning to provide students with the real-world training in both engineering design and professional skills development needed while working in a team environment on an unmet need in biomedical engineering. By structuring the course in a service-learning model, student teams are provided the opportunity to conduct community outreach by getting involved in engineering projects with community partners as actual clients. Service-learning has been defined as an experiential education that allows students to apply knowledge and skills from the classroom to meet community needs through completing project,^19^ which in our case is focused on finding biomedical engineering solutions to clinical problems in the community and reflecting on those experiences. This framework allows the students to reflect on the importance of developing their professional skills along with practicing engineering design through the lens of community engagement. Implementing service-learning into biomedical engineering design courses also directly prepares students in developing technologies that address real public health needs, and thus allows students to directly communicate with community partners as end users for continuous validation throughout their design projects. In the following teaching tip, we introduce this approach to utilizing service-learning experiences for first-time biomedical engineering design students in a sophomore-level course in order to help address curriculum gaps in professional skills development. In addition, we describe our strategy in carrying out the service-learning modality to obtain community-sourced, clinically-relevant projects that nurture students’ interest and confidence in engaging the community for identifying and addressing needs with genuine societal impact.

## NOVEL INITIATIVE

### Overview of Initiative

Enhancing professional skills development has been emphasized as a foundational need for engineering graduates entering the biomedical workforce. Traditionally, biomedical engineering curricula are heavy in technical and analytical skills development with little integration of professional skills development aside from capstone senior design courses. To deal with this challenge, we created a service-learning, team-based design course for sophomore level engineering students to cultivate their professional skills while synergistically providing real-world biodesign experiences early on in their academic training. The innovative aspect of using the service-learning framework in biomedical engineering design courses is allowing students to reach out to clinics, hospitals, and other partners in the community to identify unmet needs to serve as the basis for their engineering design projects. Rather than a volunteer experience, the structured interaction of service-learning between the students and the community partners is setup to allow the students to serve as teams under a professional setting working to design, build, and test an engineering solution to meet the needs of clients’ proposed problems. This experiential approach inherently fosters improvements in students’ professionalism in regards to organizational skills, communicating as a team, writing, as well as confidence in interacting with clients and end-users during needs assessment and device validation. The student’s role in reaching out to local clinics and hospitals to identify their community partner is an important aspect that allows students to gain experience in communicating and engaging the needs of the community. Students benefit in taking true ownership of their design projects and this service-learning framework enables a significant amount of independent learning outside of the classroom through professional relationships with their community partners.

### Rationale for Initiative

The rationale for implementing this approach is based on practices of team-based learning experiences with real-world client-sourced problems that have shown to build students’ professional skills.^7^ In having students identify community-partners as the clients and designing engineering solutions to their unmet clinical needs, students enhance their sense of social responsibility by applying technical knowledge to the benefit of society while also enhancing their professional competencies. Taking part in open-ended problem solving with a client gives students the opportunity to practice engineering design while exercising prioritization, teamwork, organization, and communication in order to create a device which meets the client’s needs. Asking the students to take the initiative in identifying community needs as well encouraging students to reflect on the community partners perspectives of their designs allows students to gain professional skills that may not be exercised in other courses.

There are several learning environments that try to engage students in building professional skills that mimic those to be encountered when joining the biomedical workforce. Observational studies have shown that engineering design projects help in preparing engineering students with professional skills when the experience is as close to participating in authentic engineering practice.^13,16^ Efforts to provide professional skills training through engineering design courses often fall into two categories: (1) student-framed and user-oriented projects; or (2) client-framed and student-driven projects. Reports have highlighted that student-framed projects while being effective in flexing student creativity do not provide the same extent of professional experience and often result in devices that are not as practical/useful as compared to client-framed project.^7^ In contrast, client-framed problems while being more conducive to developing professionalism have been reported to not hold students’ interest as much as student-framed projects.^4,7^ Our rationale is therefore to encompass the benefits of these approaches to increase the students’ interest and ‘ownership’ of the project by having them solicit community clinics and hospital units for unmet needs and allowing the student teams to decide upon which client-framed project to take on for their design projects. We believe this approach is supported by existing observations that professional skill competencies can be keenly developed through ‘real-world’ experiences^10^ and when using learning environments that are most authentic such as when students connect and work together with clients to mimic their future professional engineering practice.^1^ The implementation of this novel initiative is thus in line with observed findings that client-framed, student-driven projects provide the greatest authenticity of the engineering design experience for building professional skills. Moreover, by supporting student autonomy in having students initiate interactions with community clinics and hospitals to identify unmet community needs with engineering solutions, students feel greater ownership of the project and intrinsically solve the issue of sourcing real-world problems by making those external connections as they would in a professional setting. Through allowing student teams to select their own clinical partners, we aim to motivate students in working within areas that they naturally have more empathy and thus inspire them in this initial service-learning experience to grow their sense of social responsibility.

### Implementation of Initiative

**FIGURE 1.**
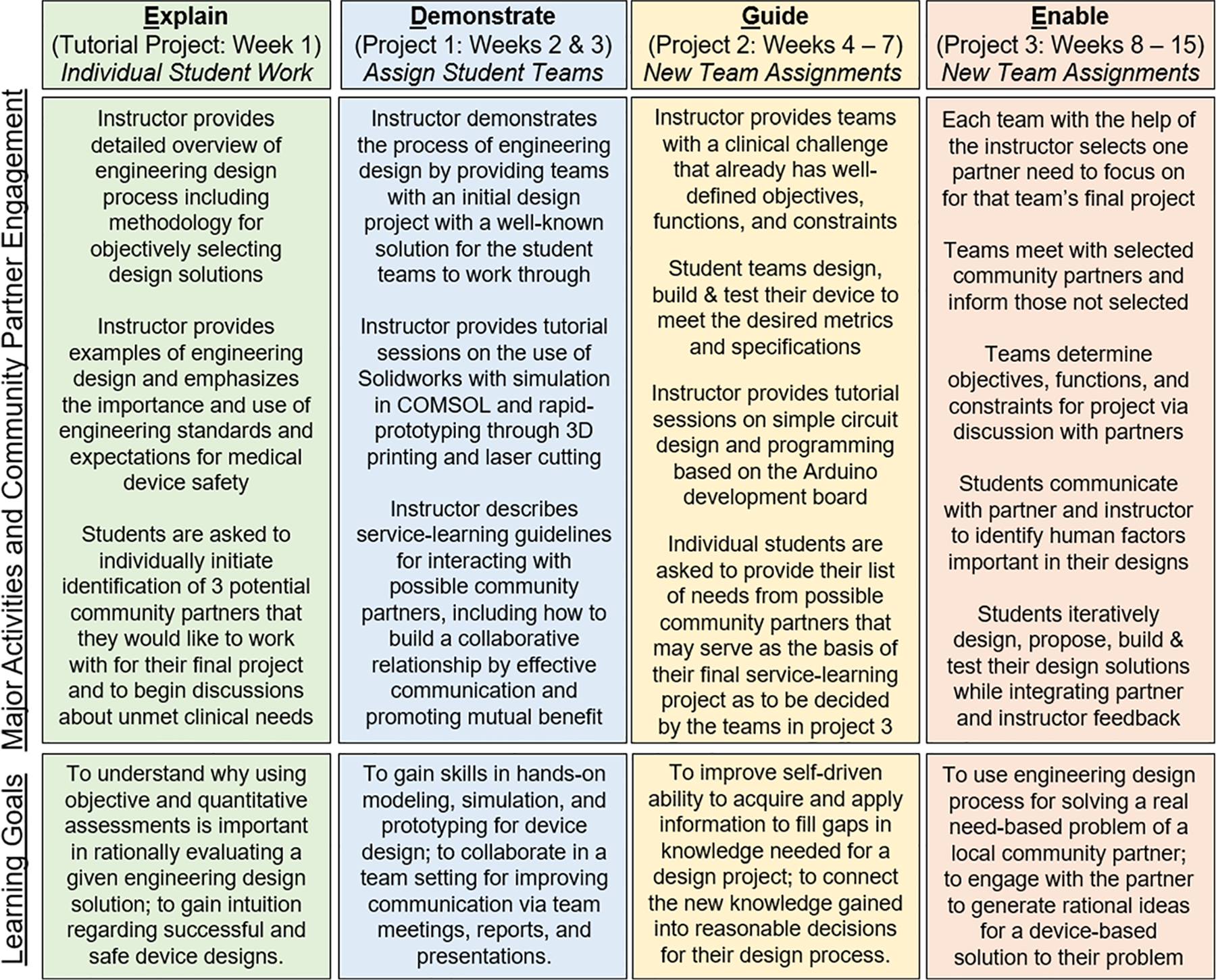
Overview of sophomore BME design course layout. An initial introduction to engineering design is provided in week 1, Following in week 2, students undergo a two week project walking them through the engineering design process with a well known solution (project 1). In week 4, students are guided through the engineering design process in a four week project to design, build, and test their own solution to a provided problem (project 2). In week 8, student teams work independently to identify a need from a community partner for their service-learning project and move forward to design, build, and test a prototype while incorporating community partner feedback in the process. These three consecutive projects provide student teams with an increasing level of autonomy in their role in the engineering design process culminating in the service-learning project (project 3) with real community partner clients.

In implementation of the sophomore service-learning design course, the instructor follows the classic job instruction training method^6^ also known as EDGE (explain, demonstrate, guide, and enable) for the overall course structure in first providing an introductory overview to the entire engineering design process in week 1, conducting a worked example of the engineering design process with a real-life example in weeks 2 and 3 (project 1), allowing students to work out an engineering design process with a provided need with guidance from the instructor in weeks 4 through 7 (project 2), and finally allows students to identify a need from a community partner to conduct their own independent team-based design project in weeks 8 through 15 which is their service-learning project (project 3). The layout of the course is structured as shown in Fig. 1. Each of the projects follow the model of: (1) understanding the problem and relevant domain knowledge, (2) design, (3) prototyping, (4) evaluating, and (5) iterating for improvement. The student teams’ primary mentorship and guidance comes from the instructor, specifically providing training about the engineering design process, foundational and technical knowledge for the respective design projects, guidance on how to interact with community partners, as well as connecting the teams with campus resources for device fabrication and testing. It is recommended for each student group to work with a separate community partner for their service-learning project. The community partners do not serve directly as mentors but rather serve as “clients” to take part in discussions with the corresponding student groups to inform them of clinical needs and assist in providing feedback for design validation. The community partner also provides the instructor with regular assessment of the group’s professionalism in their interactions. Community partners that have not previously participated in our program undergo a vetting process in the form of a meeting with the course instructor to make sure the partner is aware and agree to the expectations for serving as a “client”, how they will ultimately assess the final project of the student teams, as well as what they can expect from the student design teams. Community partners and student groups when paired are thus made aware of the importance of having frequent discussions as well as the benefit to the student teams in interacting with potential end users as to enable device designs that better fit the users’ needs. The instructor must conduct periodic follow-ups to ensure that the students and community partners are satisfied with their interactions and if necessary, allow the instructor to navigate any challenges. Accountability of the student team members as well as enhancing students’ critical thinking relies on student teams assessing each other based on the quality of their device design, prototypes, and testing. Allowing this assessment inherently encourages students to reflect more on how they are performing in those parts of the design processing. Anonymous internal assessment of team member contributions also provides a mechanism to reflect inwardly on their weaknesses and provide students to be accountable for their amount of effort put forth with their respective team.

In carrying out this course, it is important for the instructor to put forth every effort to convey a strong sense of professionalism regarding the client-based projects as to validate the importance of the service-learning project and substantiate its authenticity as corresponding to real-world professional engineering practice as would be conducted in the workplace. Engineering students benefit most in solving real-world problems corresponding to those that they may meet when joining the workforce.^2,9^ To encourage professionalism, students should be asked to reflect on how they may best demonstrate professionalism through their communication, teamwork, and organization. For the example of communication, professionalism may be conveyed through their attitude, body language, choice of words, and demonstrated respect for the community partners opinions as well as respect for their time. For monitoring professionalism, students may be made aware that their partners assessment of their professionalism as well as their professionalism during group presentations are a component of their team grade. In the final service-learning project, instructors should serve more like facilitators to mimic the role of a manager of the engineering design teams as they work toward creating a device to solve their identified problems together with input from the external clients. As such, the instructor primarily intervenes in the project only to ensure safety or keeping the project and design continually moving in the right direction in terms of focusing on addressing the needs statement.

### Community Partners and Approval of Service-Learning Project

Identification of community partners for the service-learning design course is predominantly through the individual students; however, if the institution has a service-learning or community outreach center with a list of partnering organization then it is encouraged to make the students aware of any clinically relevant groups that have already partnered with the university. The instructor informs the students in the beginning of the semester that they should identify three potential community partners with clinical needs that may benefit from an engineering solution in the form of a simple device. Later in the semester (week 8) when students are in their project 3 teams, the students discuss which of the community partner and corresponding project their team prefers and propose this project in discussions with the instructor. Here the instructor should weigh several factors before approving the students proposed first choice of community partner project, including the following considerations: the relevance to biomedical engineering; the feasibility of designing, building, and testing a prototype in the given timeframe and within the capability of the student team; the required budget and access to resources to complete the project; and if it encompasses elements of design and prototyping that parallel what was taught in the course. The next step in this process involves the instructor communicating with the proposed community partner based on the contact information provided by the students. Here the instructor will define clearly the expectations of both the student teams (including timeline, capabilities, and any limitations) and also the community partner (availability and willingness to provide assessment of the students’ design ideas and follow up with the instructor each week). This is also a good opportunity to validate the project needs from the partner as well as clarifying that this is only an 8 week engineering design project. Assuring a reliable point of contact for the student and instructor to interact with at the organization is important and confirming that the partner is willing to commit to providing evaluation of the student projects is particularly important for new community partners that the instructor is not yet familiar. Once approved, the students are asked to follow up weekly with the community partner throughout the design process in order to allow for continuous verification of their user-centered designs. By giving the students the freedom to choose which community partners to work with, this approach can give the students a greater feeling of project ownership and allow them to select community partners with which they feel more connection.

## REFLECTION

In this teaching tip, we introduced an approach to improve students’ professional skills development by a service-learning experience to work on client-based problems from community partners that closely mimics professional engineering design team situations they may experience when entering the workforce. This sophomore course is the first experience that our biomedical engineering undergraduates have with open-ended design projects. As such, students early-on in the course feel a mixture of excitement of applying their knowledge in a new way as well as hesitation due to the ambiguity in finding a “right” answer as they learn to embrace the idea of iterating to improve devices that do not perform to their metrics and specifications on the first try. In reviewing the students periodically throughout the course, we have found important trends in the students learning experience and professional skills development including teamwork from the perspectives of effort vs collaborative experience. Below we highlight ways in which the experiential service-learning projects proved successful as well as provide suggestions for implementation based on our experience with this course format.

### Considerations Prior to Implementation

Some considerations before carrying out such experiential learning projects are as follows. Primarily, student teams are best served if provided with access to physical spaces aside from a traditional classroom that is more conducive for meetings with access to computers as well as workbench space for prototyping. In addition, access to resources outside of the classroom including fabrication and rapid prototyping equipment is essential for the realization of the students designs. Students can be hesitant initially about reaching out to community partners outside of the university, it is therefore beneficial to provide an overview of service-learning along with example ‘scripts’ for how students should reach out to potential clinical partners professionally. Reports from students that the course provide high reward but high demand were common thus timing of the course within the curriculum may be an important consideration to prevent further overloading of students in any particularly ‘busy’ semester. In order to maintain an effective learning environment, it is recommended to have course sections split up into sizes of no more than 28 students, such that the instructor oversees no more than seven groups of four students each. Alternatively, additional instructors or graduate teaching assistants are recommended to assist in guidance, advice, and troubleshooting if attempting larger cohorts.

### Considerations During Implementation

Intentional randomization of student team members with efforts for minimization of overlapping team member across the three sequential projects throughout the semester allows the students to experience working with different individuals and practice the phases of teamwork within a variety of teams. The choice of assigning new teams at the beginning of each of the three distinct projects was found to provide the benefit of connecting students that normally would have few opportunities to interact, as this is one of the first biomedical engineering courses in the curriculum thereby providing the needed opportunity to introduce them to peers in the same major. By making the teams a non-permanent assignment, students were able to practice resolving intra-team tensions across different team experiences. Changing the teams for each project also provides the instructor a means for longitudinally tracking the peer-assessment of individual team members when in different groups; thereby providing greater confidence when assessing the extent of contribution by each team member. Such peer assessment is recommended, as it allows students to reflect not only on what their team-mates had contributed but also thinking more deeply about their own contributions to each project. In becoming aware of how their participation will be perceived by the group, it was said to be a motivating factor.

The instructor is encouraged to follow up initially with the community partners selected by the student teams for the final service-learning project to ensure the reported needs statements of the teams are congruent with the unmet clinical need of the community partner. Following up weekly with the community partner is encouraged to ensure that the student teams are staying engaged with the partner throughout the design process. This can take the form of a brief phone call or email the partner to inquire about their perception of the student teams’ level of professionalism including teamwork, communication, and organization as well as providing an opportunity to assess the partners satisfaction or concerns. This provides not only a point for student assessment but allows for quickly identifying any issues.

It is suggested during the three distinct team-based projects that more detailed guidance be provided at the beginning to give way to sequentially more autonomy and exploration by the third project which is the service-learning project. The course leader in project 1 will provide students with the understanding for how to effectively go through the engineering design process and what categories of design consideration are important to be aware. As student teams move on to the project 2, they are given more independence in their design, building, and testing approaches having gained more training by this time on various prototyping, materials, as well as important design controls. In project 3, the design teams are now fully independent in their design and development of a device to meet their community partner’s clinical need while being under the supervision of the instructor as more of a facilitator. By project 3, students are aware of the expectations and common troubleshooting techniques that they will have for project 3, as well as the process flow for which they should carry out a design project. By the end of project 3, it is reasonable to expect the teams’ prototypes to have undergone testing with at least one design iteration toward meeting the desired metrics and specifications; however, the extent to which those prototypes meet the design goals will vary depending on the complexity of the project and the skill level of the students within the team for the respective project. While project success does have merits, because the course is focused on collaboration in a team setting, it is designed to improve communication skills through group meetings, presentations, and written reports as well as professional interactions with their community partners.

### Evidence of Successful Implementation of the Initiative

**FIGURE 2.**
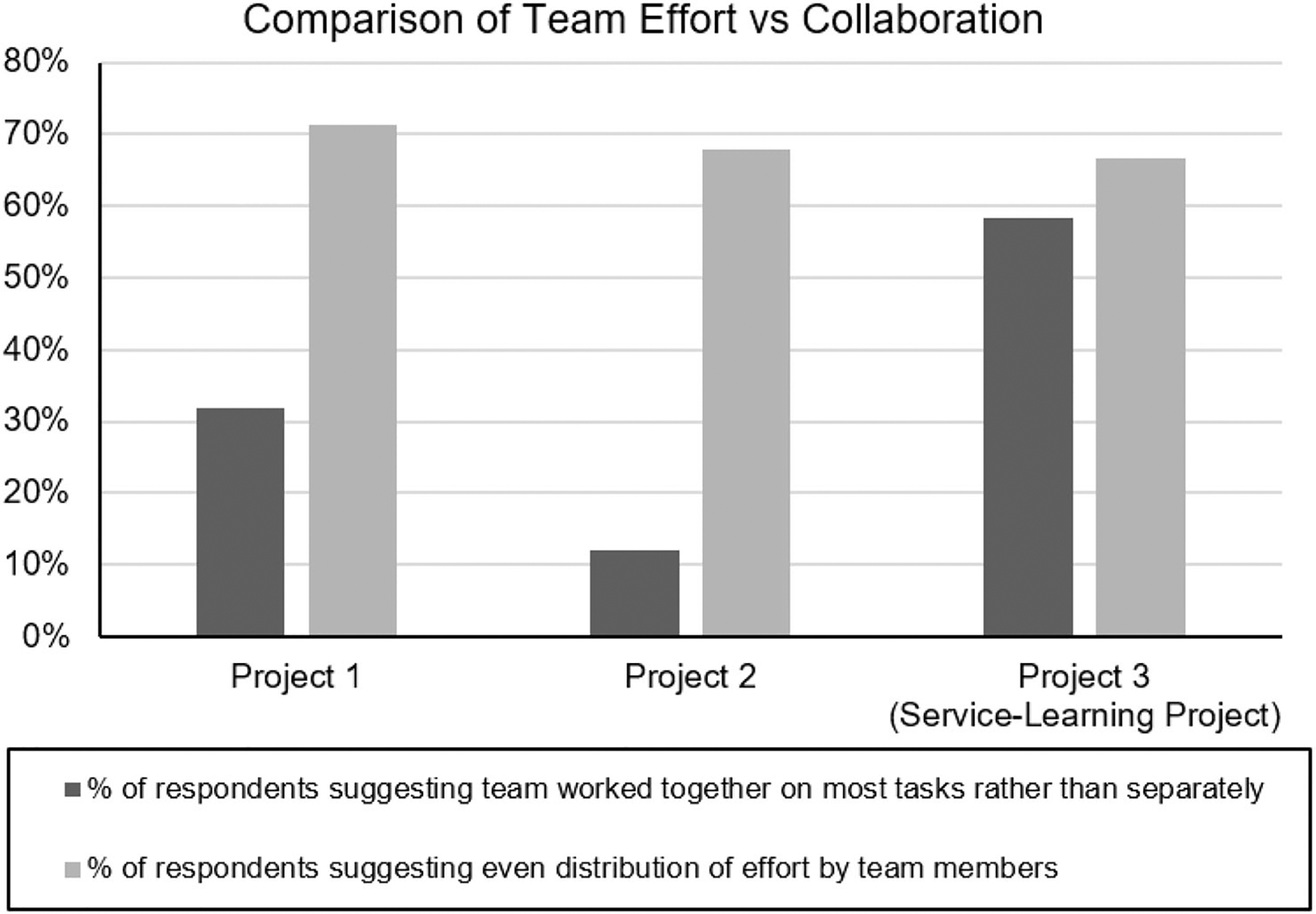
Survey results throughout the phases of the course to assess the extent of team member efforts as compared to collaborative teamwork after each of the three design projects with the final project 3 being the experiential service-learning project with a community partner ‘client’.

At the onset, the student reflections were never intended for reporting in a journal and as such we may only provide a summarization of the common themes along with observed trends. As this is among the first classes that biomedical engineering (BME) undergraduates take together only with other BME students, the feedback from the students was overwhelming that this class helps to support a sense of comradery and by requiring the use of fabrication facilities across the campus allowed the students to get a sense of their campus resources and community. Comparing student surveys across the three projects, we could clearly see that student worked collaboratively on tasks together as a team as opposed to division of labor and this was to a greater extent in the service-learning project when compared to the first two instructor-guided projects (Fig. 2). Based on the responses, the extent of contributed effort from team members did not appear to change much as the semester progressed to projects with more autonomy. While additional data would be needed to confirm this hypothesis, we suggest that the Fig. 2 reports of greater collaboration in projects 1 and project 3 may have been attributed to the students being put into entirely new experiences and seeking support through collaboration with their teammates for project 1 and 3. Project 1 represented their first ever engineering design project, and the new experience of service-learning and interacting with clients in project 3 may have encouraged more group interactions and intra-team support. Project 3 represented the first time the students were able to experience service learning and thus may have a greater desire for teamwork and collaboration given the unfamiliar territory. Surveying the students on their confidence in working on a design project, 95% of respondents answered affirmatively that they are confident in their ability to carry out a design project after the second project, and 100% of respondents answered affirmatively after the third project. This may highlight the importance of these first two projects in preparing students prior to their autonomy in the service-learning experience of project 3.

From additional surveys on the impact of this course, we found that this experience provided students with an entrepreneurial mindset and as suggested that this course made them overwhelmingly more likely to want to form a start-up company (75% of respondents), as compared to 8% that were less likely to want to form a start-up company, and 17% that were unaffected by the course experience in their likelihood of forming a start-up company. From the open-ended question of what skill sets do you feel you gained most from this service-learning project, student responses predominantly included themes of the following key areas: design; communication; persistence; problem solving; responsibility; working together on a team; planning and executing a plan efficiently; thinking critically; an appreciation for organization within dynamic groups; and learning independently. The surveys discussed above were previously conducted for program evaluation; thus, in consultation with the University of Texas at Arlington’s Office of Regulatory Services, the previously collected survey data was deemed to be exempt from IRB approval as it was in alignment with program evaluation and to improve the bioengineering program.

## CONCLUSIONS

In this teaching tips article, we discuss a sophomore BME design course incorporating experiential service learning with community partners as clients. Our findings support it to be effective in developing the student’s skills in engineering practice and encouraging professional skills development through collaborative teamwork and interaction with community partners as clients. The service-learning projects enhanced their ability to communicate and reflect on their interpretation of the project progress as well as practice teamwork and organizational skills in a professional setting. This course structure also was effective for sourcing projects with real-world clinical impact and that allowed students to feel ownership of their projects. Overall, the students’ interaction with their community partners throughout the design process facilitated a uniquely authentic engineering experience for the BME undergraduates that ultimately helped them develop their professional skills while practicing biomedical device design.
